# A case of Bainbridge-Ropers syndrome with breath holding spells and intractable epilepsy: challenges in diagnosis and management

**DOI:** 10.1186/s12883-022-02573-w

**Published:** 2022-02-16

**Authors:** Tuba Rashid Khan, Alison Dolce, Kimberly Goodspeed

**Affiliations:** 1 Department of Pediatrics, Division of Child Neurology, University of Texas Southwestern Medical Center, Dallas, TX USA; 2 Department of Pediatrics, Division of Pediatric Neurology, Emory University School of Medicine, Atlanta, GA USA

**Keywords:** Bainbridge-Ropers syndrome, Breath-holding spells, ASXL3 gene

## Abstract

**Background:**

Bainbridge-Ropers syndrome is caused by monoallelic ASXL3 variants on chromosome 18. Clinical features include dysmorphic facies, developmental delay, intellectual disability, autistic traits, hypotonia, failure to thrive, seizures and hyperventilation. Breath-holding spells with choreathetoid movements have been previously described.

**Case presentation:**

We describe an 11-year old boy who has daily intractable seizures reported since birth, developmental delay, autistic features and feeding difficulties. He was eventually found to have de novo, heterozygous pathogenic variant (c.1612G > T, p.E538*) in the ASXL3 gene. He has frequent episodes of breath-holding accompanied by dystonic posturing with right leg extension and head turning without ictal EEG correlate. The breath-holding spells have been refractory to several medication trials including iron supplementation, acetazolamide, and desipramine.

**Conclusions:**

This case represents a more severe phenotype of Bainbridge-Ropers Syndrome than previously described with refractory breath-holding spells with dystonia, intractable epilepsy, and progressive cerebral/cerebellar atrophy. Breath-holding spells cause significant morbidity, are poorly understood, and have very limited treatment options.

## Background

Bainbridge-Ropers syndrome (BRPS) is caused by monoallelic ASXL3 variants on chromosome 18 first reported in 2013 [1]. ASXL3, together with ASXL1 and ASXL2, functions to regulate gene expression as a protein complex that acts as a histone methyltransferase [1]. The phenotypic spectrum includes dysmorphic facial features, developmental and intellectual disability, autistic traits, hypotonia, failure to thrive, and epilepsy [2]. Additionally, episodes of hyperventilation have been reported, as well as a case of hyperventilation-induced athetosis in a 10-year old patient with ASXL3 deficiency, intellectual disability, and vision impairment [3]. To our knowledge, this is the first case of refractory breath holding spells with dystonia in a patient with BRPS. Further, our patient demonstrates progressive cerebral and cerebellar atrophy as well as an epileptic encephalopathy.

## Case presentation

Our patient presented at 2 years of age with global developmental delay, autistic traits, feeding difficulties and paroxysmal events concerning for seizure. He was born via uncomplicated full-term vaginal delivery. He had hypersomnolence in infancy and spells of crying with stiffening or tremor of his extremities. He had multiple initial normal EEGs at an outside center with one showing occipital dominant photoparoxysmal responses; however, clinical events were never captured on EEG and they did not improve with a trial of Zonisamide. At 6-years old he had his first atonic seizure described as behavioral arrest with head bobbing and loss of truncal tone with quick recovery followed by brief unprovoked laughter and smile. The events were associated with generalized, high-amplitude, 3–3.5 Hz notched delta frequency epileptiform discharges (often with an occipital lead-in). Interictal spikes were also seen over the left centro-parietal regions. Seizures occurred up to 140 times daily at onset and were refractory to numerous medications. Subsequent EEGs were all abnormal with variable background slowing and multifocal epileptiform discharges (commonly maximal over the left centro-parieto-temporal regions) consistent with an epileptic encephalopathy. His epilepsy remains intractable on oxcarbazepine, rufinamide, clobazam, and cannabidiol. Zonisamide, clonazepam, topiramate, lacosamide and valproic acid were tried and discontinued due to lack of effectiveness and side effects.

On exam, he has a high arched palate, hypertelorism, low set ears, and left exotropia. He is hypotonic and walks with a broad-based gait. He has bilateral rigid pes planovalgus deformities of both feet secondary to calcaneonavicular and subtalar coalition that are managed with orthotics. Polysomnography revealed mild to moderate obstructive sleep apnea, but he was not deemed a candidate for tonsillectomy or adenoidectomy. Initial genetic testing including karyotype, chromosomal microarray, genetic epilepsy panel, and Fragile X testing were non-diagnostic. At age 10-years, whole exome sequencing completed in a CLIA-certified lab identified a de novo, pathogenic, heterozygous non-sense sequence variant in the ASXL3 gene. The genetic change is a single nucleotide change at c.1612G > T (p.E538*), within exon 11 (NM_030632) that yields a premature stop codon predicted to cause nonsense mediated decay and are typically deleterious in nature [4]. In accordance with ACMG Guidelines, this is a likely positive test as he has a pathogenic variant and clinical phenotype consistent with BRPS.

**Fig. 1:**
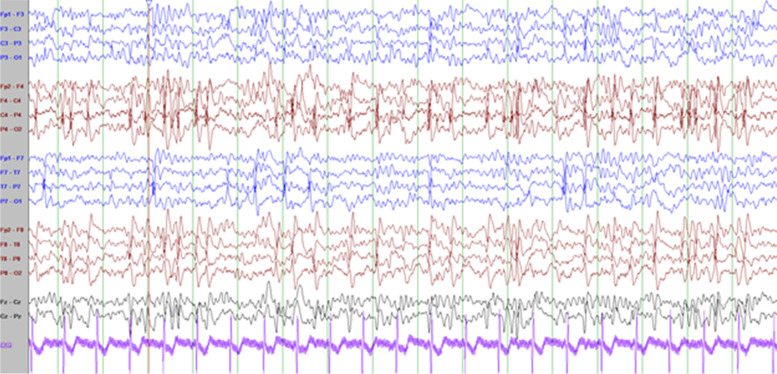
Interictal background: Frequent, near continuous, high amplitude spikes occurring over the right centrotemporal > left temporoparietal regions superimposed on a slow background (Sensitivity at 15 uV/mm)

**Fig. 2:**
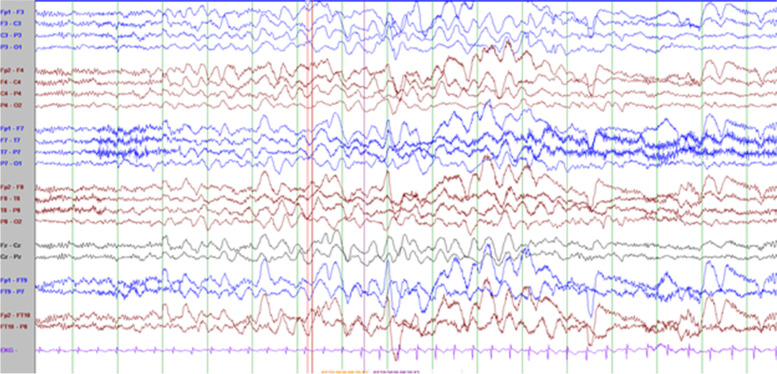
Breath-holding spell characterized by burst of high amplitude polymorphic theta-delta slowing (bifrontal maxima) with no epileptiform discharges. Patient noted to have loud audible expiration 6 s in to the slowing (Sensitivity 10 uV/mm)

**Fig. 3:**
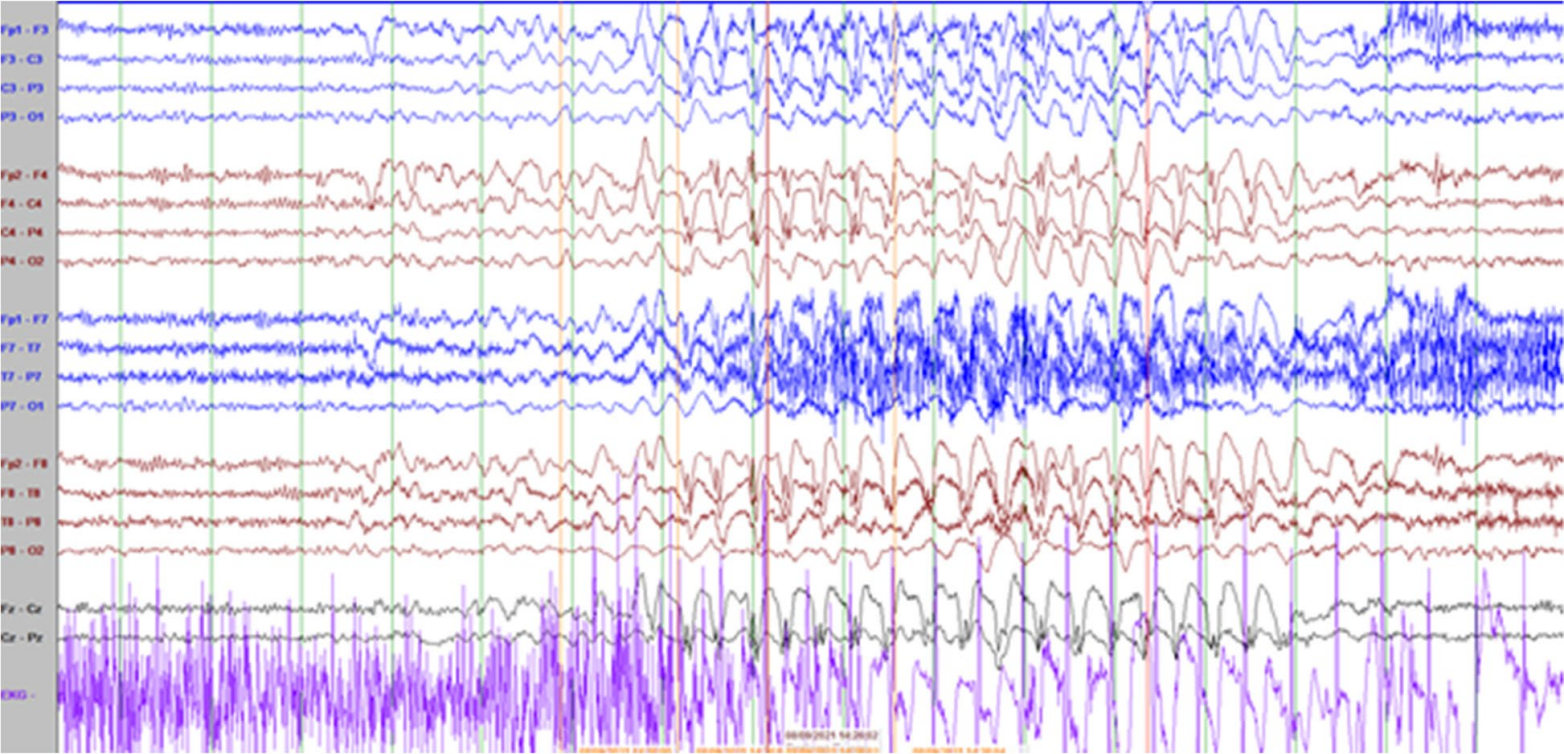
Electroclinical seizure characterized by generalized, high amplitude, bifrontal maxima, 2–2.5 Hz spike and slow wave activity associated with behavior arrest and head bobbing (Sensitivity 10 mV/mm)

**Fig. 4:**
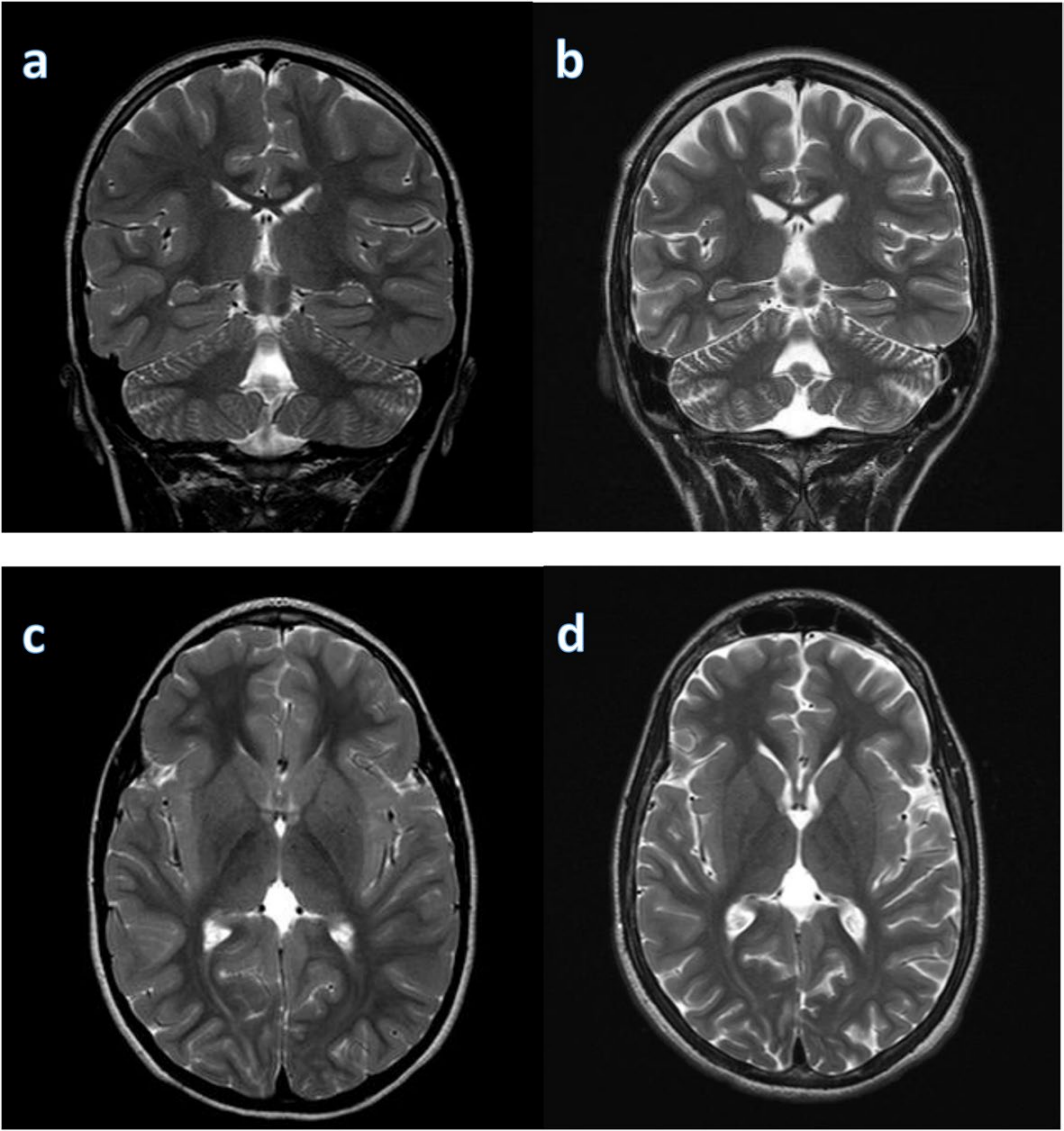
Longitudinal MRI brain assessment. MRI T2 image showed progressive cerebral and cerebellar atrophy with increasing ventricular size over the 5 year period from age of 6 years (**a**, **c**) to age of 11 years (**b**, **d**)

He has frequent daily episodes of non-pallid, non-cyanotic breath-holding accompanied by dystonic posturing with leg extension and head turning that disrupt his activity and often lead to falls that were first noted at the age of 9-years. These events have been captured on long-term EEG monitoring and noted to be associated with high amplitude polymorphic delta slowing following an audible expiration without any associated epileptiform discharges (Fig. 2; with reference to inter-ictal EEG in Fig. 1). His electroclinical seizures, in comparison, are described as behavioral arrest with full body shaking and sometimes a head turn to the left which are associated with left temporal spike and slow wave discharges. (Fig. 2) cannot be definitely ruled out as a possible seizure because though the patient starts to hold his breath before the slowing starts and 6 s in he exhales and there doesn’t seem to be an EEG correlate at onset of event, it could still be scalp negative at onset. The breath-holding spells have been refractory to several medication trials including iron supplementation, acetazolamide, and desipramine. At 11-years old he had severe clinical deterioration with sudden weight-loss of 13-lb over one month with progressive lethargy, encephalopathy, and decreased oral intake. He was found to be hyperammonemic to a maximum of 189 micromol/L. This was presumed to be related to valproic acid use (maximum level in the preceding week was 119.5 mcg/mL). At that time he also had near continuous spike-wave discharges on his EEG, concerning for non-convulsive status epilepticus (Fig. 3). He had also been given a recent trial of acetazolamide for breath-holding spells. His two initial brain MRIs at age 2-years and 6-years were unremarkable, but during this period of clinical decline, repeat imaging revealed cerebral and cerebellar atrophy (Fig. 4). His decompensation was ultimately attributed to a combination of adverse effects of valproic acid and acetazolamide. Valproic acid was discontinued around this time due to hyperammonemic encephalopathy. He was recently re-started on zonisamide and has had some reduction in the frequency and duration of these events but has continued to demonstrate worsening balance and disruptive sleep–wake patterns.

## Discussions and conclusions

This case represents a more severe phenotype of BRPS than previously described with refractory breath-holding spells with dystonia and intractable epilepsy. Breath-holding spells cause significant morbidity, are poorly understood, and have limited treatment options available. In addition, although epilepsy is associated with ASXL3 variants, intractable cases to this degree are not described. Breath-holding spells are thought to be associated with autonomic dysfunction and cause cyanotic or pallid syncopal spells that could lead to a status epilepticus or hypoxic convulsions [5]. This may potentially aggravate the underlying epileptic pathophysiology in our patient although direct evidence would be warranted. In our patient, captured electroclinical seizures were not typically preceded by a breath holding spell as these were two distinct events. Whether the breath-holding spells are unrelated to or in fact a part of the disease process will also need further observation. Brain imaging is typically normal in patients with BRPS, although cortical atrophy and hypoplasia of the corpus callosum and cerebellar vermis have been reported [6]. One possible cause of cerebral and cerebellar atrophy could be related to long-term use of valproic acid, which has been reported to be reversible after discontinuation of valproic acid [7]. The latest brain MRI of our patient was acquired around the time when valproic acid was discontinued due to hyperammonemia and future follow up brain scans will help to clarify if it is reversible due to medication use or part of the underlying disease process. It is also plausible that his clinical deterioration could be associated with his underlying genetic epilepsy syndrome. A recent report suggests that if truncation in exon 11 is located upstream of base c.2985 or codon p.(995) (only 55 bps away from the last exon/intron junction), then pathogenicity is expected to be very strong and associated with nonsense decay [8]. The nucleotide substitution in our patient at c.1612G > T would meet this criteria. Our case appears to be atypical in comparison to previously reported BRPS cases with other truncating mutations because he has a more severe epileptic encephalopathy and refractory breath-holding attacks with dystonia that lead to falls compared [2]. Schirwani et al. [8] reported a truncating variant in c.1606C > 7, p.(Glu536*) in exon 11, which is in close proximity to the variant in our patient; however, information regarding disease severity in their study is not available. Although we believe that disease progression in our patient is related to the de novo *ASXL3* pathogenic variant, other concurrent and contributing processes that made the disease more severe cannot be ruled out. Given his medically refractory epilepsy, consideration may be given to other treatment modalities including the ketogenic diet, VNS and the possibility of a pre-surgical evaluation to determine if he could be a candidate for a focal resection versus a palliative surgical procedure such as corpus callosotomy.

To our knowledge, this is the first case with progressive cerebral and cerebellar atrophy correlating with a decline in neurologic function. This case expands the known BRPS phenotypes with a case of progressive epileptic encephalopathy and breath-holding spells with dystonia and falls as well as obstructive sleep apnea and orthopedic complications, representing a more severe phenotype than previously reported.

## Data Availability

Not applicable.

## Abbreviation

BRPS: Bainbridge-Ropers syndrome
